# Hydrogen Bond Dynamics and Phase Transitions of Water inside Carbon Nanotubes

**DOI:** 10.3390/nano13020284

**Published:** 2023-01-10

**Authors:** Amit Srivastava, Jamal Hassan, Dirar Homouz

**Affiliations:** 1 Department of Physics, Khalifa University of Science and Technology, Abu Dhabi 127788, United Arab Emirates; 2 Department of Physics, University of Houston, Houston, TX 77030-5005, USA; 3 Center for Theoretical Biological Physics, Rice University, Houston, TX 77030-1402, USA

**Keywords:** carbon nanotubes, water, hydrogen bond dynamics, molecular dynamics simulation

## Abstract

Water dynamics in nanochannels are altered by confinement, particularly in small carbon nanotubes (CNTs). However, the mechanisms behind these effects remain unclear. To address these issues, we carried out extensive molecular dynamics (MD) simulations to investigate the structure and dynamics of water inside CNTs of different sizes (length of 20 nm and diameters vary from 0.8 nm to 5.0 nm) at different temperatures (from 200 K to 420 K). The radial density profile of water inside CNTs shows a single peak near the CNT walls for small nanotubes. For CNTs with larger sizes, water molecules are arranged into coaxial tubular sheets, the number of which increases with the CNT size. Subdiffusive behavior is observed for ultranarrow CNTs with diameters of 0.8 nm and 1 nm. As the size of CNTs increases, Fickian diffusion becomes evident. The hydrogen bond correlation function of water inside CNT decays slower than in bulk water, and the decay rate decreases as we increase the diameter of the CNTs. In large CNTs, the hydrogen bond lifetime of the innermost layer is shorter than the other layers and depends on temperature. Additional analysis of our results reveals that water molecules along the CNT axis show a non-Arrhenius to Arrhenius diffusion crossover. In general, the diffusion transition temperature is higher than that of bulk water, but it depends on the size of the CNT.

## 1. Introduction

The structure and dynamics of confined water in nanochannels are significantly different from those in bulk [1,2,3,4]. These characteristics have great potential in a large number of applications such as drug delivery [5,6], intercellular water transport [7], cancer treatment [8], and many other important applications [9,10,11]. Research on the diffusion of water molecules in artificial water channels is of interest to many researchers because it can lead to new designs of synthetic channels with similar performance to biological water channels. In recent years, water in carbon nanotubes (CNTs) has been studied extensively using different experimental techniques [12,13] and molecular dynamics simulations [14]. These methods extracted valuable information, such as the temperature dependence of water diffusion in nanohydrophobic confinements, which is of great interest in many applications.

Due to the confinement and hydrophobic interactions, water molecules inside CNTs behave differently than in bulk water. The effects of nanoconfinements on the structure and dynamics of water molecules have been addressed mainly through computer simulations. Hummer et al. [15] demonstrated that water molecules inside CNTs of diameter from 0.68 nm to 0.81 nm are arranged as a single-file water chain. They also revealed the properties of confined water are dramatically affected by the diameter of the CNTs. Pascal et al. [16] performed MD simulations of water–nanotube systems of different diameters and revealed that the water inside CNTs is more stable than the bulk and also that water dynamics depend on the CNT diameter. Mukherjee et al. [17] have shown that the mean square displacement (MSD) of water molecules confined in short open-ended (6, 6) CNTs depends weakly on the length of the nanotube, and the diffusion behavior is Fickian due to the presence of strong hydrogen bonding between adjacent water molecules. The same research group [18] also demonstrated that water molecules confined in a narrow carbon nanoring with no open ending exhibit a single-file diffusion where MSD∼$t^{1/2}$. We recently [19] reported on the water dynamics inside different CNT sizes and temperatures using both two-dimensional NMR diffusion relaxation *D*-$T_{2eff}$ spectroscopy and MD simulations. We found that water molecules inside CNT resolve in two or more tubular components, acquiring different self-diffusion coefficients that depend on the size of the CNTs.

Moreover, confined water in CNTs exhibits multiple phase structures and phase transitions. Noon et al. [20] found that by tuning the CNT diameter, the nanoconfined water shows a liquid–solid critical point at which liquid and solid coexist. Mukherjee et al. [17] also predicted a solid-like ordering structure for water molecules inside CNT (6,6) under ambient conditions. Using different water models, Kumar et al. [21] observed the solid-like ordering structure inside long CNT(6,6). This structure arises due to a stronger hydrogen bond (HB) between the adjacent water molecules. Confinement also affects the intrinsic dynamics of the HB and the orientation of the dipoles of the water molecules. The number of HBs can form and break depending on the environment, or they can be broken upon the deformation of the nanotube wall. In addition, the number of HBs can increase or decrease depending on the interaction with the atoms that form the nanostructures.

The confined water inside CNTs adopts distinct organizations to maximize the number of HBs. Thus, the number of HBs might change inside CNTs. These changes in the HB network structure are expected to change the melting point of water. Using MD simulation, Takiawa et al. [22] reported nine distinct ice types in several zigzag CNTs of different diameters. Later, Chiashi et al. [23] measured the melting point of water confined in CNTs with 16 different chiralities, with diameters ranging from 0.95 to 1.26 nm, and they observed that the melting point was close to the bulk in most of the studied CNTs. They also reported that the same type of ice in different diameters of nanotubes may have different melting points. The question that then arises is whether the melting temperature changes depend on the size of the nanotubes.

Another puzzling behavior of water in a small nanotube is the breakdown of the Stokes–Einstein relation between diffusivity and viscosity in supercooled and confined water, an anomaly in diffusion coefficients that implies the existence of two diffusion regimes, separated by a temperature above which fragile-to-strong (FTS) transition occurs [24,25]. Diffusion of strong liquid follows Arrhenius, whereas a fragile liquid has a super-Arrhenius (or non-Arrhenius) behavior. Liquid can be divided into these two categories, but water is an exception. Water is fragile at ambient temperature, while it appears to be strong upon supercooling [24]. Many studies have been carried out to explain the FTS transition in water but, among all of them, two of the hypotheses are the most relevant, proposed by Angel and coworkers [26] and Stanley et al. [27]. Angel and coworkers associated the FTS transition in water with glass transition temperature, whereas Stanley et al. treat water as being two liquids in one. Recently [28], we examined the fragility of water molecules confined inside CNT with different sizes using the SPC/E [29] water model. We used the Speedy–Angell power law parameter to explain the dynamic behavior of water. We found that for small and large CNT size the water confined inside CNTs are similar to bulk water, whereas in 3.0 nm CNT size, the fragility parameter value deviates from those of bulk water. This indicates the high fragility of the water molecules in this system and is also consistent with our previous experimental study.

It is important to point out that although MD simulations provide a great deal of information about the structure and dynamics of confined water, results may vary if different water models were used. Due to the anomalous properties of water, no single water model can reproduce the physical properties of water over the whole range of thermodynamic states. Kumar et al. [21] computed the structural and thermodynamic properties of water molecules confined inside the narrow CNTs of diameter 0.81 nm using five different water models: TIP3P, modified TIP3P, SPC/E, SPCFw (flexible water model), and POL3 (polarized water model). They found the SPC/E water model is the optimum choice for the study of water confined inside CNTs.

Many MD simulation studies [28,30] were devoted to investigating the diffusion of confined water molecules inside CNTs of different sizes. Mostly, simple water models such as SPC/E [29] and 4-site such as TIP4P models [31] were used in these studies, and often the investigation was carried out at room temperature. Therefore, the effect of temperature on water diffusion inside CNTs has not been explored extensively yet. It is known that upon increasing CNT’s sizes, the ordering of confined water changes from a one-dimensional (1D) chain to coaxial water tubular sheets (CWT). An important question is to examine and find the effect of temperature and water models on the water ordering inside large CNTs. To address these open questions, we systematically studied the effect of temperature on confined water diffusion inside CNTs at temperatures ranging from 200 K to 420 K. We used the TIP3P water model to study the water ordering inside CNTs of different sizes and compared our result with the SPC/E water model. In particular, we analyze how confinement affects the intrinsic dynamics of the hydrogen bond network in confined water molecules. We further examine the fragile-to-strong transition observed in water.

## 2. Materials and Methods

MD simulations at a constant number of particles, pressure, and temperature were performed to investigate the diffusion coefficient of water confined inside CNTs of different sizes. The TIP3P model was used because it agrees well with the experimental results such as specific heat, density, etc., and also predicts both structural and thermodynamics properties of water compared with other water models. The obtained results were compared with another highly investigated water model, SPC/E.

**Figure 1:**
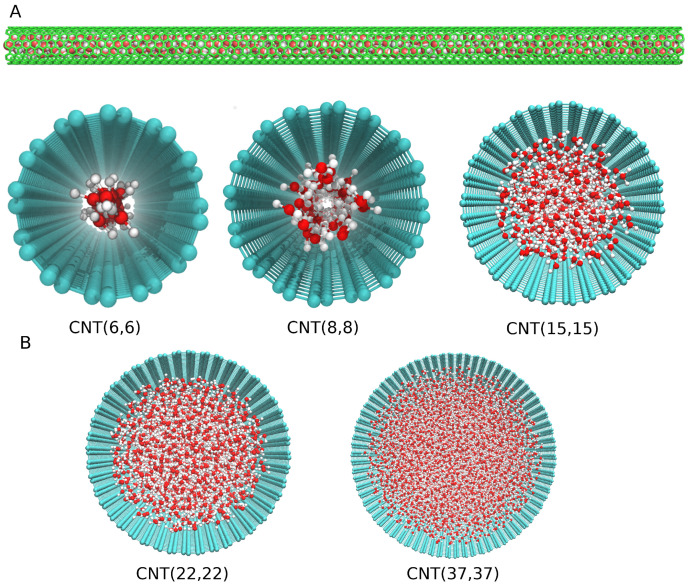
Schematic diagram of a water–CNT system used in simulation: (**A**) the side view of (8,8) CNT filled with water and (**B**) top views of different diameter CNTs filled with the water molecules. Oxygen, hydrogen, and carbon atoms are shown in red, white, and green colors.

Water molecules were confined inside the CNT with different diameters, and chiralities were simulated. The (n, m) notation is used to characterize the chirality of CNTs. We investigated five armchair carbon nanotubes (n = m), namely (6,6), (8, 8), (15, 15), (22, 22), and (37, 37). The diameter of the nanotube as a function of *n* and *m* index is given as:

(1) $$d\;=\;\frac{\sqrt{3}}{\pi }a\sqrt{n^{2}+m^{2}+nm},$$

where a = 1.418 Å is the C-C bond length. The CNT diameters corresponding to these indices are 0.8 nm, 1 nm, 2 nm, 3 nm, and 5 nm, respectively. The initial configuration of the water–CNT system is shown in Figure 1, with the diameter of CNTs considered ranging from 0.8 to 5 nm. The length of the CNT is set to 20 nm.

### 2.1. Simulation Setup

The MD simulations were performed using NAMD [32]. Water molecules in the simulations were represented using the TIP3P model [33], which is known to accurately predict many of the bulk properties of water. The nonbonded interactions between the carbon atoms were modeled using Lennard–Jones Potential. We choose the parameters given by Werder et al. [34]. The CNTs were frozen throughout the simulations by putting a positional restraint which fixed the positions of the carbon atoms throughout the simulations. The water confined inside CNT was investigated in the range of temperature from 200 K to 420 K. Temperature was incremented by 10 K from 200 K to 300 K, then by 20 K steps up to 420 K. Langevin thermostat was used to set the temperature to the target value. The pressure was kept constant at 1 bar using Nose–Hoover Langevin piston with a period of 100 fs and a damping time scale of 50 fs. Unlike previous MD simulations, where CNT is infinitely long along the CNT axis, here, in our simulation, we allowed water molecules to flow in and out of CNTs with finite lengths. The length of CNT in our simulation is 20 nm. Long-range electrostatic interactions were computed by the Particle Mesh Ewald summation method (PME). A timestep of 2 fs is used for the integration of the equation of motion. Bonded interactions were calculated every time step, while nonbonded interaction was calculated every two steps, with a cutoff of 12 Å and a switching function of 10 Å. All the simulated systems were minimized for 10,000 steps, then gradually heated to the target temperature, and then equilibrated at this temperature for 50,000 steps (100 ps) before the production runs. The production simulations were run for a total time of 50 ns. The system configuration was saved every 500 steps (1 ps) into the output trajectory for further analysis.

Several quantities were calculated to address the pertinent question related to the effect of confinement on water molecules inside the CNTs. The radial distribution function was calculated to identify the possible melting point of the system. We also computed the axial diffusion coefficient to describe water mobility. These quantities will help us to determine whether the system is in a liquid or solid state.

### 2.2. Radial Distribution Function

It is known that the sharp decrease in the peak of the radial distribution function is a sign of phase transition [35]. To quantify the possible transition temperature, we computed radial distribution functions of water molecules confined inside the CNT, which are defined as:

(2) $$g\left(r\right)\;=\;\frac{1}{N}\sum_{i=1}^{N}\sum_{j=1,i\neq j}^{N}<\delta \left(r_{ij}-r\right)>$$

where N is the total number of water molecules and $r_{ij}$ is the distance between the *i*th and *j*th molecules along the x–y axis. We used the oxygen atom of the water molecule to determine the distribution functions.

We also computed the radial density profile for water inside each simulated system to elucidate the ordering of water molecules. The detail of radial density calculation is given in our previous publication [28].

To characterize the structure of the water molecule inside the CNT, we calculated the number of hydrogen bonds (HBs) per water molecule. We used the following geometrical criteria for HBs:

$$\alpha \leq 30^{\circ }$$

$$|r_{\mathrm{OO}}|\leq 3.50Å$$

where α is the OH···O angle, and $|r_{\mathrm{OO}}|$ is the distance between two oxygen atoms.

To examine the arrangements of water molecules inside CNT, in the XY plane, density maps are calculated where the water oxygen atom is used. The density map was obtained by dividing the corresponding plane into square bins of 0.1 Å length and then counting the number of oxygen atoms in each square bin. Higher oxygen densities are shown in red, while low oxygen densities are shown in blue.

### 2.3. Diffusion Coefficient

Due to confinement and hydrophobic effects, water diffusion in the radial direction inside CNTs is minimal and can be ignored [30]. Consequently, we consider only the axial diffusion coefficient ($D_{z}$) along the *Z*-direction. The value of $D_{z}$ was determined using the mean squared displacement function (MSD) in the axial direction, which was calculated using

(3) $$\begin{array}{ccc} \langle \Delta z^{2}\left(t\right)\rangle & \;=\; & \frac{1}{N}\sum_{i=1}^{N}{\langle {\left[z_{i}\left(t+t^{\prime }\right)-z_{i}\left(t^{\prime }\right)\right]}^{2}\rangle }_{t^{\prime }}\\ \lim_{t\to \infty }\langle \Delta z^{2}\left(t\right)\rangle & \;=\; & 2dD_{z}t^{\alpha } \end{array}$$

where *t* is the time difference, $t^{{}^{\prime }}$ is a time origin, and *N* is the number of water molecules. *d* denotes the number of dimensions considered. The time exponent α specifies the self-diffusion mode for water molecules inside the CNT. If the time exponent equals 1, the water molecule undergoes Fickian diffusion in the CNT, such that MSD scales linearly with time. Water molecule undergoes single-file diffusion when α = 0.5, whereas α = 2.0 corresponds to ballistic diffusion. However, when 1 <α< 2, the diffusion mode is superdiffusion. MSD computation for confined water requires extensive averaging. MSD was calculated over a time interval of 1.0 ns at a sampling rate of 1.0 ps. MSD was then averaged over 50 such time intervals. The MSD of the confined water cannot be computed for arbitrarily long times, due to the limited duration of stay of the water molecules inside the nanotube—since the ends of the nanotube are open, the confined water molecules exchange their positions with the water molecules in the outside reservoir. The interval length 1.0 ns was chosen carefully to give water molecules enough time inside CNTs before exiting. To estimate the diffusion constant $D_{z}$, we used our recently proposed algorithm, which is capable of fitting with different regimes corresponding to different time scales [36]. The algorithm fits the MSD to a continuous piece-wise function and finds the breakpoints that separate different modes of motion. The overall diffusion coefficient of water inside the CNT was calculated in addition to the diffusion coefficients of individual density profile components.

### 2.4. Hydrogen Bond Correlation Function

Dynamic and thermodynamic properties of water depend on the nature of hydrogen bonding. Therefore, it is important to obtain detailed information on HBs of water at different temperatures and sizes of CNTs. To study the formation-breaking process of the HB network, we calculated the HB autocorrelation function (HBACF) using the following geometrical criterion:

(4) $$C_{HB}\left(t\right)\;=\;\frac{<h(t+\tau )\cdot h(\tau )>}{<h{\left(\tau \right)}^{2}>}$$

where h(τ) is the HB order parameter. If the two tagged water molecules form HB at time τ, the variable h(τ) = 1, otherwise h(τ) = 0. The HBACF decays completely after only a few picoseconds. HBACF measures the probability of a pair of water molecules being bonded at time τ and remaining bonded at time t+τ, while ignoring possible breaking within that time frame. In other words, the HBACF provides insight into how persistent an HB network is. The geometric criteria for hydrogen bond selection are already presented in the previous subsection. A biexponential function was fitted in HBACF to obtain the parameters for a short-time ($\tau_{1}$) and long-time ($\tau_{2}$) decay of the autocorrelation function.

(5) $$C_{HB}\left(t\right)≅A_{1}e^{\left(\frac{-t}{\tau_{1}}\right)}+\left(1-A_{1}\right)e^{\left(\frac{-t}{\tau_{2}}\right)}$$

Finally, the lifetime of the hydrogen bond is calculated using the Equation (6):

(6) $$\tau_{HB}\left(t\right)≅A_{1}\tau_{1}+\left(1-A_{1}\right)\tau_{2}$$

where $\tau_{HB}$ is HB lifetime.

## 3. Results and Discussion

### 3.1. Radial Density Profile Shows Water Arrange in Coaxial Water Sheets

**Figure 2:**
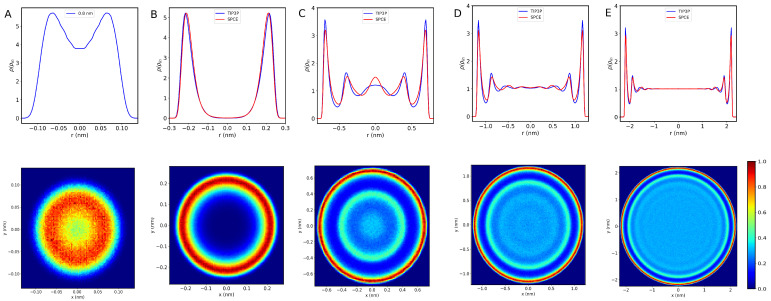
Radial local density of water inside different CNT sizes (**A**) 0.8 nm, (**B**) 1.0 nm, (**C**) 2.0 nm, (**D**) 3.0 nm, and (**E**) 5.0 nm at room temperature. The x-axis represents the inner diameter of CNT, where zero represents the center of the nanotube. The density map of water molecules inside various CNT sizes is shown below the density profile of each CNTs. High water density regions are shown in red color, while low water density regions tend to have a dark blue color.

First, we analyze the structure of water inside different sizes of CNTs. The ordering of the water molecules inside the CNTs depends on the confinement size. For ultranarrow CNTs (d∼0.8 nm), water molecules form a one-dimensional polymer-like chain. As we increase the diameter of CNTs, more water fills the empty space inside the CNT and forms coaxial water tubular sheets (CWT). Figure 2 (lower panel) shows the density map corresponding to the oxygen occurrence during MD simulation for five CNT systems with diameters of 0.8, 1.0, 2.0, 3.0, and 5.0 nm at room temperature (300 K). From these results, we see that water arranges in a 1D polymer chain inside the CNT with a 0.8 nm diameter. Upon increasing the CNT diameter, water starts to transform into the CWT structures. These structures are shown in green and red color in density maps. The observed CWT sheets are in agreement with the previous work (see, for example, reference [14] and references therein). The number of CWT sheets depends on the size of CNTs, oxygen–oxygen, and oxygen–carbon interactions. It is also observed that the number of CWT sheets increases with CNT diameter. Furthermore, the dynamics of water molecules at the center of CNTs, in larger sizes, approach the bulk limit. Moreover, it is observed that the layered structure of water molecules in the simulated systems does not depend on the temperatures.

It is known that confinement alters the dynamics of water inside CNTs, as indicated by the calculated water density profiles. The effect of a layered structure as described above can be seen from the radial density profile. Figure 2 (upper panel) shows the radial density distribution of water molecules using two different water models (SPC/E and TIP3P) inside different sizes of CNTs, at room temperature. The value of *r* = 0.0 corresponds to the center of CNT. The figure clearly shows water arranged in a single-file chain structure inside CNT with a 0.8 nm diameter. On the other hand, inside 1.0 nm CNT, water molecules form a layered structure. The single peak in the density profile implies that water forms a single tabular layer, in agreement with previous work [37]. Due to hydrophobic interactions, the density profile of water molecules inside CNTs becomes sharper, and the peak moves closer to the CNT walls.

Upon increasing the CNT diameter, additional water layers are formed corresponding to multiple peaks in the density profile. For large CNTs (3.0 nm and 5.0 nm, for example), a bulk-like density profile is observed near the center of the CNT [14,37]. In 3.0-nm CNT, we observe two additional layers consistent with the NMR experiment reported by us previously [19]. In general, there is a good agreement between the results obtained from the two used water models and those reported earlier.

### 3.2. Self Diffusion Coefficients

**Figure 3:**
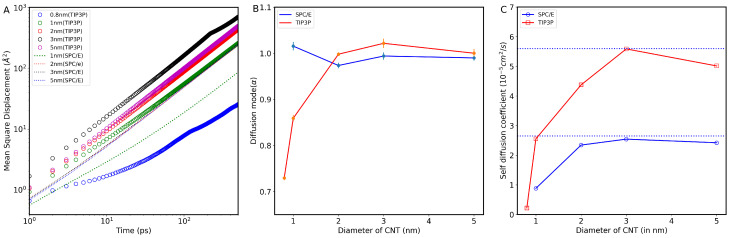
(**A**) Time dependent of mean squared displacement (MSD) of water inside different CNT sizes, at room temperature. (**B**) Variation of α, the exponent that defines the diffusion mechanism shown in the equation, as a function of the CNT diameter. (**C**) Self-diffusion coefficients versus CNT diameter. Results were obtained using two different water models discussed within the text.

To gain more insight into the effect of CNT structure on water molecules’ mobility, we computed the self-diffusion coefficient of water molecules inside different CNT sizes at different temperatures. We find a direct connection between water structure and diffusion. In addition, the confinement effect may result in water adopting multiple diffusion modes. Figure 3A depicts the logarithmic plot of the MSD curve versus time for water molecules in the axial direction, z-axis, inside CNTs, at 300 K. To obtain these results, two water models (TIP3P and SPC ) were used for water inside 1.0, 3.0, and 5.0 nm, while only one model (T1P3P) was used for the 0.8 nm CNT. It is observed that MSD behavior in 0.8 nm CNT size is different from those inside larger CNT sizes. At the initial time stage, 0.8 nm CNT water molecules exhibit a single-file behavior consistent with the previous studies [15]. As expected, MSD values increase with increasing the size of CNTs, reaching the maximum values inside 3.0-nm CNT. Figure 3B shows the diffusion modes of water inside CNTs using the two water models.

The identification of the diffusion modes is based on the time exponent coefficient parameter (α, where α = 1 corresponds to Fickian diffusion) as in Equation (3). To obtain these results, we used the fitting algorithm defined in [36]. In the algorithm, a flexible model is fitted to the MSD data that allows for different behaviors at various time scales. There are two regimes in the model: short and long time, separated at $\tau =\tau_{1}$. Based on the SPC/E water model, we found that the diffusion modes of water for all CNT sizes are Fickian. Results from the SPC/E water model are in agreement with those published previously [38]. On the other hand, with the TIP3P model, water inside 0.8-nm CNT shows a subdiffusive mode, where MSD∼$\tau^{0.5}$ for $\tau <\tau_{1}$) and Fickian mode for $\tau >\tau_{1}$. The subdiffusive behavior might indicate single-file water diffusion, as previously suggested by [15,18].

For the TIP3P model, α increases with the pore size of CNTs. We note that the time exponent coefficient increases from α∼0.73 for the CNT with a diameter of 0.8 nm, to α∼0.86 for the CNT with a diameter of 1 nm, and α∼1 for the CNT having a diameter 2 nm. This indicates a transition from single file to normal diffusion. Therefore, in a nutshell, water molecule experiences the subdiffusion mode in the CNT with pore sizes 0.8 nm and 1 nm and eventually converges to the Fickian diffusion in CNTs with pore sizes larger than 2 nm. Furthermore, it looks like using different water models generates different values of parameters related to water dynamics, in agreement with the literature [21,39]. Consequently, one should take into account these variations when results from different publications are compared.

Based on the MSD plot versus time, we accordingly calculated the axial self-diffusion of confined water inside different diameters of CNTs. Figure 3C shows these results for different CNTs at 300 K using the two water models. The dashed line in the figure denotes the self-diffusion coefficients of bulk water, namely 2.53 ($10^{-5}$ cm^2^ s^−1^) obtained using SPC/E [21] and 5.32 ($10^{-5}$ cm^2^ s^−1^) [39] obtained using TIP3P. As the diameter of CNT reaches 3.0 nm, the diffusion coefficients approach the bulk value, obtained by the models, for both data sets. The results agree with the previous studies [37,38,40,41], which showed that water inside large CNT sizes that accommodate more than two layers loses the memory of the CNT wall and tends to acquire the bulk water structure. Therefore, in the large CNT nanopores, the density of water molecules at the central region of the CNT resembled that of the bulk phase [14]. At 300 K, it is noted that diffusion reaches its maximum value in 3.0 nm CNT and then decreases slightly beyond this size. This matches qualitatively with experiments, where Gokura et al. [19] also observed the enhancement in water diffusion for CNT with a diameter of 3.5 nm using the two-dimensional nuclear magnetic resonance diffusion–relaxation ($D-T_{2eff}$) spectroscopy method. The observed diffusion coefficients in experiments agree qualitatively with our MD simulation results. The possible mechanism for water diffusion enhancement at certain CNT sizes might be related to the competition between the water–wall contact area and the volume occupied by each water molecule.

**Figure 4:**
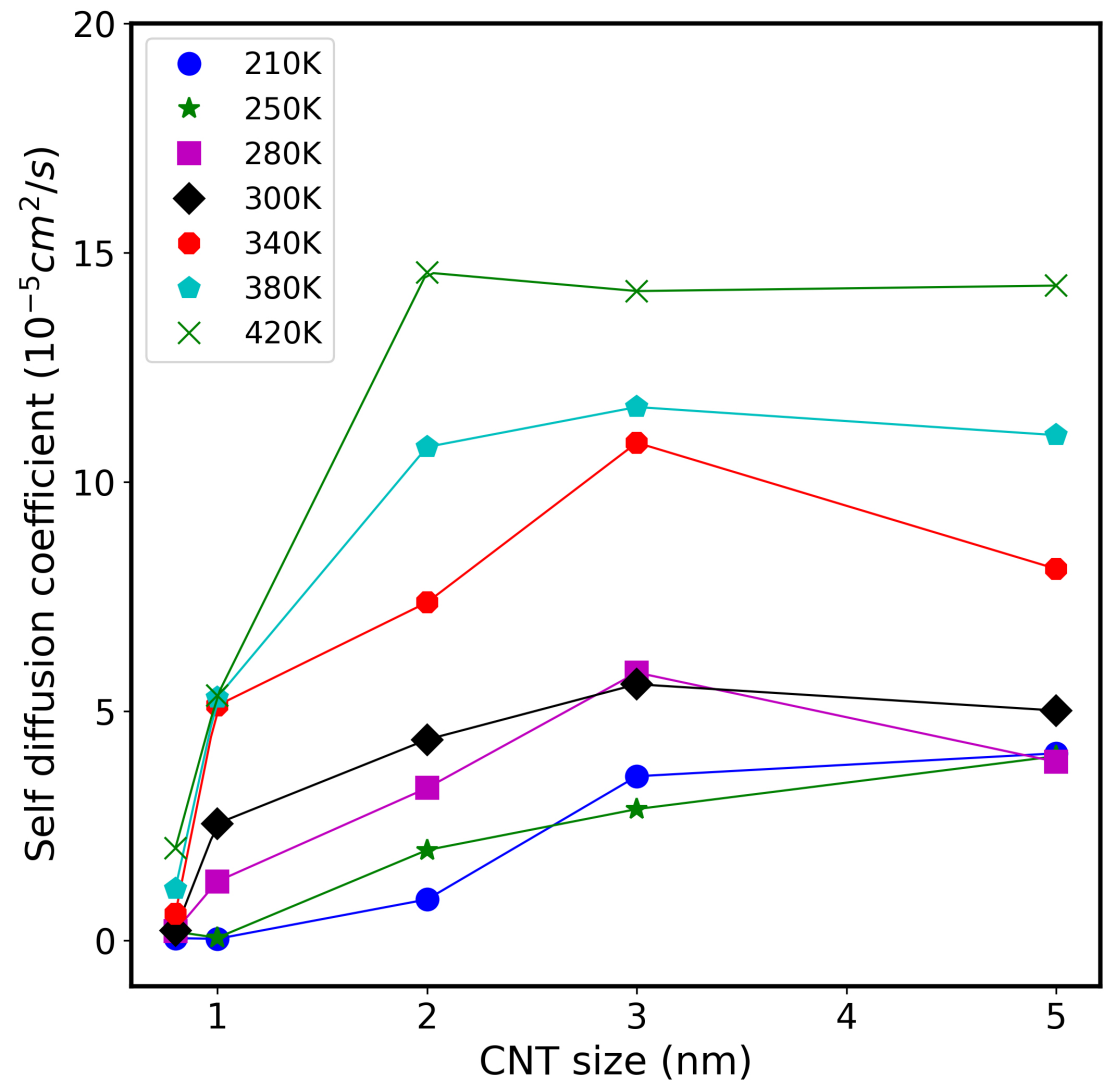
Self-diffusion coefficient of water molecules inside CNT of different sizes, at temperature range between 210 K to 420 K, using the TIP3P model.

To examine the role of temperature on the dynamics of confined water inside CNTs, we calculate the axial self-diffusion coefficient $D_{z}$ for different CNT sizes and different temperatures. Figure 4 shows these results at different temperatures ranging from 210 K to 420 K. As expected, the values of $D_{z}$ of water increase with temperature for most systems. For example, the value of $D_{z}$ for 3 nm CNT nanopore is 3.58 × $10^{-5}$ cm^2^ s^−1^ at 210 K, 2.84 × $10^{-5}$ cm^2^ s^−1^ at 250 K, 5.84 × $10^{-5}$ cm^2^ s^−1^ at 280 K, 5.59 × $10^{-5}$ cm^2^ s^−1^ at 300 K, 10.86 × $10^{-5}$ cm^2^ s^−1^ at 340 K, 11.63 × $10^{-5}$ cm^2^ s^−1^ at 380 K, and 14.16 × $10^{-5}$ cm^2^ s^−1^ at 420 K. In addition, the value of $D_{z}$ for ultranarrow CNT (diameter 0.8 nm) increases from 0.0395 × $10^{-5}$ cm^2^ s^−1^ at 210 K, to 0.147 × $10^{-5}$ cm^2^ s^−1^ at 250 K, to 0.207 × $10^{-5}$ cm^2^ s^−1^ at 280 K, to 0.343 × $10^{-5}$ cm^2^ s^−1^ at 300 K, to 0.576 × $10^{-5}$ cm^2^ s^−1^ at 340 K, to 1.126 × $10^{-5}$ cm^2^ s^−1^ at 380 K, and to 2.007 × $10^{-5}$ cm^2^ s^−1^ at 420 K. Above 300 K, even for the small 1.0 nm CNT, the diffusion of water reached (∼5.1 × $10^{-5}$ cm^2^ s^−1^). It is important to emphasize that the TIP3P model gives bulk water $D_{z}$ of about 5.0 × $10^{-5}$ cm^2^ s^−1^, as seen in the previous figure. This means that the obtained values of water diffusion using the TIP3P model are overestimated in comparison with the reported experimental data.

Finally, we see that water diffusion depends on its layered structure, as shown in Figure S1 (in the Supporting Information document), which shows the values of $D_{z}$ for different water sheets inside various CNT systems at temperatures ranging from 210 K to 340 K. The diffusion coefficient tends to increase as we move away from the CNT wall for systems with more than one CWT, in agreement with experimental results [19].

### 3.3. Hydrogen Bond Dynamics

**Figure 5:**
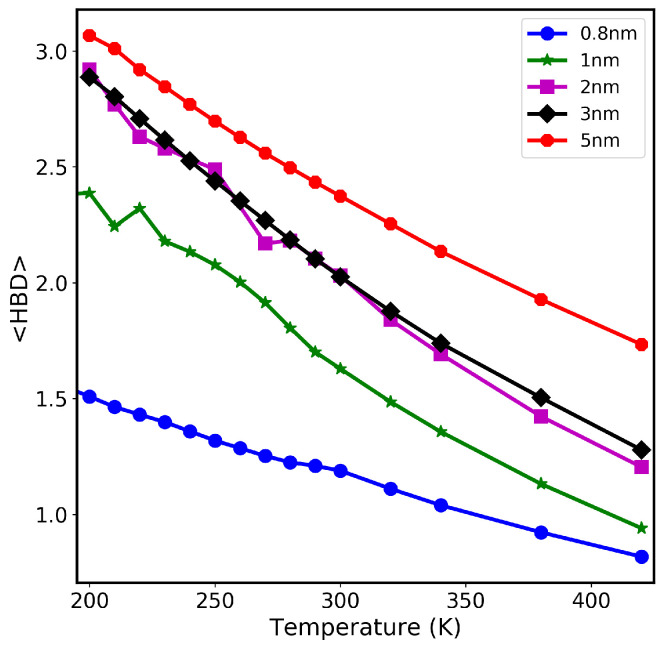
Average number of hydrogen bonds per water molecule versus temperature in different CNT sizes.

To understand the role of confinement in determining the self-diffusion of water molecules, we investigate the average number of HBs per water molecule. The definition of HBs between water molecules is already described in the Section 2. Figure 5 shows the average number of HBs per water molecule for the water–CNT system of sizes ranging from 0.8 nm to 5 nm at different temperatures (from 200 K to 420 K). As seen from the results, upon increasing temperature, the number of HBs of confined water decreases in all the CNT sizes considered in this study. This is expected as water becomes more mobile and loses its HB network structure. Moreover, at a given temperature, the number of HBs per water molecule increases with increasing the confinement size. For example, at 300 K, the number of HBs per water molecule in 0.8 nm is 1.19, then increases to 1.63 in 1.0 nm, reaching 2.38 in 5.0 nm. When compared with bulk water, water inside CNTs has fewer HBs due to the confinement effect and hydrophobic interactions. As a matter of fact, water near carbon atoms of CNT walls does have fewer HBs than bulk water [38].

The average number of HBs is highly dependent on the water model. The SPC/E model produces different results than the TIP3P model. For example, at 300 K, the number of HBs per water molecule in 1 nm CNT is 2.2 for the SPC/E water model, whereas for TIP3P it is 1.63. The $D_{z}$ value for 1 nm CNT at 300 K is 0.9 × $10^{-5}$ cm^2^ s^−1^ for the SPC/E water model, whereas it is 2.548 × $10^{-5}$ cm^2^ s^−1^ for TIP3P. Since the hydrogen bond network is less structured in the TIP3P water model compared with SPC/E, the water diffuses faster in TIP3P.

### 3.4. Hydrogen Bond Lifetime

**Figure 6:**
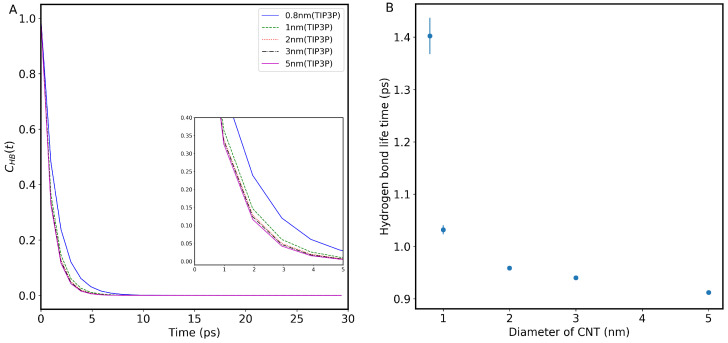
(**A**) Hydrogen bond auto-correlation function of water molecule inside different CNT sizes at room temperature. The inset shows a magnification of the data between 0 and 5 ps. **(B)** Hydrogen bond lifetime with the CNT diameter at room temperature.

To investigate the stability of hydrogen bonds among water molecules inside the CNTs, we evaluated the hydrogen bond lifetime. In this regard, the hydrogen bond autocorrelation function (HBACF) was calculated to study the effect of confinement size and temperature on the stability of hydrogen bonds. The procedure we adopted for HBACF calculation was presented in the Section 2. Figure 6 shows the variation of HBACF for different sizes of CNTs (0.8 nm, 1.0 nm, 2.0 nm, 3.0 nm, and 5.0 nm) at 300 K. As seen from Figure 6 (left panel), the HBACF decays exponentially with simulation time for all the CNTs used, but the timescale for this exponential decay changes with the confinement size. The inset of the figure clearly shows that water molecules in smaller CNT sizes retain their HB network for a longer time compared with the larger CNT channels. This is due to a large effect of nanoconfinement on the dynamics of water in small CNTs. In addition, we believe that the dominant effect here is the hydrophobic interaction between carbon atoms of the CNT walls and water molecules. Due to these effects, in small CNT sizes, it is energetically more favorable for water molecules to retain their HB network for a longer time compared with their structure inside larger CNT channels where these effects are less effective.

The hydrogen bond lifetime can be obtained by fitting the double exponential function, as described in the Section 2. Figure 6 (right panel) suggests that the hydrogen bonding network in smaller CNT sizes, on average, remains intact for a longer time compared with those inside larger confinements. This behavior might be attributed to the fact that many water molecules are close to the hydrophobic wall of the 0.8 nm CNT. Consequently, it is energetically less favorable to break and form new HBs. On the other hand, in larger CNTs, a larger portion of water molecules are at the center of the pores, and breaking and forming HB is faster on average. The hydrogen bond lifetime decreases monotonically as we increase CNT size: 1.4 ps (in 0.8 nm), 1.0 ps (in 1.0 nm), 0.96 ps (in 2.0 nm), 0.94 ps (in 3.0 nm), and 0.91 ps (in 5.0 nm CNT). As seen, upon increasing the confinement size, the HB lifetime approaches that of bulk water (0.78 ps [42]). We also observe that hydrogen bond lifetime decreases with temperature.

Water diffusivity differs among CWT sheets, as discussed above. Thus, we examine the hydrogen bond dynamics in each CWT sheet using the hydrogen bond lifetime. Table 1 shows the hydrogen bond lifetimes of water molecules corresponding to each water layer inside different CNT nanochannels at temperatures ranging from 200 K to 420 K. The table reveals the striking differences in water dynamics between the different CWT sheets. According to the data, the outermost layer of water has the longest HB lifetime, which may explain its slow diffusion. The results are consistent with previous studies and for different water models.

### 3.5. Radial Distribution Function

**Figure 7:**
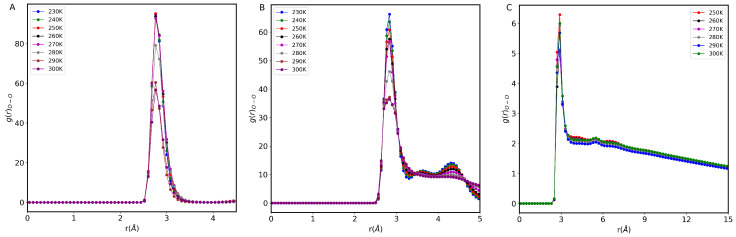
Radial distribution functions RDF of water molecules inside CNTs of different sizes. (**A**) 0.8 nm, (**B**) 1 nm, and (**C**) 3 nm. A sudden decrease in the peaks of RDF indicates a phase transition.

To analyze water phase transitions inside small confinements, we calculate the radial distribution function (RDF). It is well-known that the sharp decrease in RDF indicates the onset of a first-order phase transition. Figure 7 shows the RDF of confined water inside 0.8 nm, 1.0 nm, and 3.0 nm CNTs at different temperatures. We find that the peak position of RDF (∼2.8 Å) does not depend on temperature and remains the same for 0.8 nm and 1 nm CNTs, whereas the height of the peak decreases with temperature, dropping sharply at 270 K. When the temperature is increased further, the peak height continues to decrease until we reach 300 K and then remains constant after that. It appears that the ordered structure of the water distribution in the nanotube disappears with a sharp decrease in peak height, hence signaling a phase transition, which is usually interpreted as a fragile-to-strong transition (further details follow in the next subsection). At low temperatures, we observe two peaks in RDF for 1 and 3 nm CNTs, but their second peak position is different. In both structures, squared ice is present, but the 1 nm nanotube’s smaller diameter brings the water molecules closer together. Note that the hydrogen bond lifetime is up to 1.5 orders of magnitude larger than the bulk for confined water in 0.8 nm and 1 nm CNTs.

### 3.6. Fragile-to-Strong Transition Observed in Liquids

**Figure 8:**
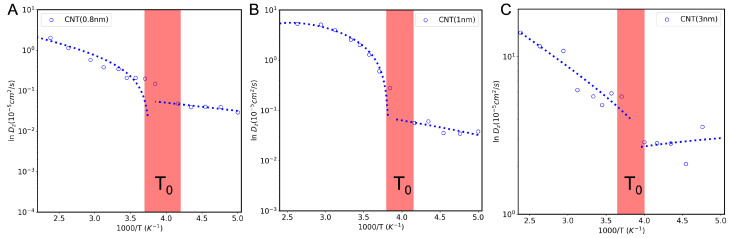
The logarithmic plot of diffusion coefficients of water versus inverse temperature inside CNTs of (**A**) 0.8 nm, (**B**) 1 nm, and (**C**) 3 nm. $T_{0}$ stands for the region in which a non-Arrhenius to Arrhenius transition takes place.

It is known that liquids are classified as strong and fragile [43]. Thermodynamic properties of strong liquids, such as the self-diffusion coefficient, obey the Arrhenius law: *D* = $D_{0}$$exp(-\Delta U/k_{B}T)$, where ΔU is the activation energy and $D_{0}$ is the pre-exponential diffusion term. On the other hand, fragile liquids obey non-Arrhenius laws. Here, we address the puzzling transition that water undergoes from fragile to strong liquid, observed in RDF analysis inside 0.8 nm, 1 nm, and 3 nm CNTs. Figure 8 shows the confined water self-diffusion coefficient versus inverse temperature inside 0.8 nm, 1 nm, and 3 nm CNTs. For the 1 nm nanotube, the diffusion coefficient approaches the bulk value at high temperatures but, at a given temperature, water diffusion decreases with decreasing nanotube diameter, as we would expect. Figure 8 shows the typical FTS transition at around *T* = $T_{0}$∼244–270 K upon cooling. The data presented in Figure 8 show a crossover from the non-Arrhenius to Arrhenius behavior, which occurs as the system approaches $T_{0}$. This crossover temperature is higher than the temperature observed for bulk water, and it does weakly depend on the CNT diameter.

## 4. Conclusions

Extensive MD simulations were carried out to investigate the size and temperature effect on the self-diffusion and hydrogen bond dynamics of confined water inside CNTs with pore sizes ranging from 0.8 nm to 5.0 nm at a temperature range between 200 K to 420 K. The effect of temperature and confinement size on hydrogen bond lifetime was also investigated very extensively. We found that the water is arranged in a one-dimensional chain in ultranarrow CNTs of 0.8 nm. By increasing the CNT size, the water molecules adopt a shell-like structure and are stratified in layers within CNTs. In addition, the number of confined water layers depends on the size of the pore. Using the TIP3P water model, we report for the first time the subdiffusive behavior of confined water inside CNTs with diameters of 0.8 nm and 1 nm. This behavior is attributed to the correlations between water–water and water–wall collisions. Water diffusion mode becomes Fickian in CNTs with pore sizes larger than 1.0 nm. Our simulations show a clear enhancement in water diffusion inside a 3.0 nm CNT. The results of the simulations show that upon increasing temperature, the HB network of the confined water weakens and increases diffusion enhancement. The hydrogen bond correlation function of water inside CNT decays slower than in bulk water, and the decay rate decreases as we increase the CNT diameter. For large CNTs, the hydrogen bond lifetime of the innermost layer is shorter compared with the other layers and depends on the temperature, too. Additionally, we found that water molecules diffuse faster near the center of large nanotubes compared with their outer shells, especially at temperatures of 280 K and 300 K for CNTs with a diameter of 3.0 nm. Moreover, the hydrogen bond lifetime is up to 1.5 orders of magnitude larger than the bulk for confined water in 0.8 nm and 1 nm CNT, which is not reported in any previous study. Analysis of the RDF and the temperature dependence of the diffusion coefficient reveals a phase transition of water inside CNTs with diameters of 0.8 nm and 1 nm. The transition is characterized by a non-Arrhenius to Arrhenius crossover behavior, which is a sign of a fragile-to-solid transition. The transition temperature is higher than that of bulk water and is dependent on pore size. We also compared the results obtained using two different water models: TIP3P and SPC/E. We found TIP3P water diffuses faster than SPC/E water due to the weaker hydrogen bond network in the TIP3P water. The results using both models are qualitatively consistent with the NMR results, although the timescale in the MD simulation is much shorter compared with that in the NMR experiment.

## Figures and Tables

**Table 1:** The hydrogen bond lifetime of water molecules is confined to the CNTs of different diameters at different temperatures. The time unit is picosecond (ps). The water shells in the table correspond to coaxial water tubular sheets formed in CNTs due to the confinement.

CNT (in nm)	Water-Shell (in Å)	Temperature (in K)													
		200 K	210 K	220 K	230 K	240 K	250 K	260 K	270 K	280 K	300 K	320 K	340 K	380 K	420 K
0.8	0–4	3.10	2.71	2.4	2.14	1.93	1.74	1.59	1.47	1.37	1.40	1.22	1.10	0.89	0.78
		(±0.06)	(±0.05)	(±0.03)	(±0.03)	(±0.03)	(±0.02)	(±0.02)	(±0.02)	(±0.02)	(±0.04)	(±0.02)	(±0.01)	(±0.01)	(±0.01)
1	0–5	3.42	3.52	2.87	2.96	2.69	2.42	2.13	1.75	1.39	1.03	0.87	0.76	0.61	0.51
		(±0.09)	(±0.13)	(±0.17)	(±0.07)	(±0.09)	(±0.07)	(±0.06)	(±0.05)	(±0.03)	(±0.01)	(±0.01)	(±0.01)	(±0.01)	(±0.01)
2	Outer-shell	-	-	2.52	2.17	1.92	1.62	1.41	1.32	1.16	0.96	0.82	0.71	0.55	0.45
	(5-10)			(±0.05)	(±0.03)	(±0.02)	(±0.01)	(±0.02)	(±0.01)	(±0.01)	(±0.01)	(±0.01)	(±0.01)	(±0.01)	(±0.01)
	Inner-shell1	-	-	2.6	2.13	1.87	1.60	1.35	1.25	1.15	0.96	0.81	0.70	0.55	0.45
	(2–5)			(±0.05)	(±0.03)	(±0.01)	(±0.01)	(±0.01)	(±0.02)	(±0.01)	(±0.01)	(±0.01)	(±0.01)	(±0.01)	(±0.01)
	Inner-shell2	-	-	2.74	2.14	1.82	1.61	1.32	1.29	1.15	0.95	0.81	0.69	0.54	0.44
	(0–2)			(±0.19)	(±0.23)	(±0.16)	(±0.04)	(±0.03)	(±0.11)	(±0.04)	(±0.03)	(±0.02)	(±0.02)	(±0.02)	(±0.02)
3	Outer-shell	3.6	2.97	2.48	2.11	1.83	1.60	1.41	1.26	1.14	0.94	0.80	0.69	0.54	0.44
	(9.8–15)	(±0.02)	(±0.02)	(±0.01)	(±0.01)	(±0.02)	(±0.01)	(±0.01)	(±0.01)	(±0.01)	(±0.01)	(±0.01)	(±0.01)	(±0.01)	(±0.01)
	Inner-shell1	3.53	2.89	2.42	2.07	1.80	1.58	1.39	1.25	1.13	0.94	0.80	0.69	0.54	0.44
	(6.4–9.8)	(±0.03)	(±0.02)	(±0.01)	(±0.01)	(±0.01)	(±0.01)	(±0.01)	(±0.05)	(±0.01)	(±0.01)	(±0.01)	(±0.01)	(±0.01)	(±0.01)
	Inner-shell2	3.51	2.87	2.41	2.06	1.79	1.57	1.39	1.25	1.13	0.94	0.79	0.69	0.53	0.43
	(0–6.4)	(±0.05)	(±0.03)	(±0.01)	(±0.01)	(±0.01)	(±0.01)	(±0.01)	(±0.01)	(±0.01)	(±0.01)	(±0.01)	(±0.01)	(±0.01)	(±0.01)

## Data Availability

The data are available upon reasonable request from the authors.

## Supplementary Materials

The following supporting information can be downloaded at: https://www.mdpi.com/article/10.3390/nano13020284/s1, Figure S1: Self-diffusion coefficients of different water shells, shown in Figure 2 of the main text, inside different CNT sizes of (A) 0.8 nm, (B) 1.0 nm, (C) 2.0 nm, (D) 3.0 nm, and (E) 5 nm. The x-axis is the distance of water shells from the center of CNTs. Colors represent different temperatures; Blue (210 K), red (250 K), green (280 K), black (300 K), and cyan (340 K).
