# Supplementary material for: Hydrogen Bond Dynamics and Phase Transitions of Water inside Carbon Nanotubes
Source: Nanomaterials (Basel). 2023 Jan 10;13(2):284. doi: 10.3390/nano13020284 (PMC9866512; doi:10.3390/nano13020284)
Supplement: Supplementary file 1 [file nanomaterials-13-00284-s001.zip › nanomaterials-2119606-supplementary.pdf]

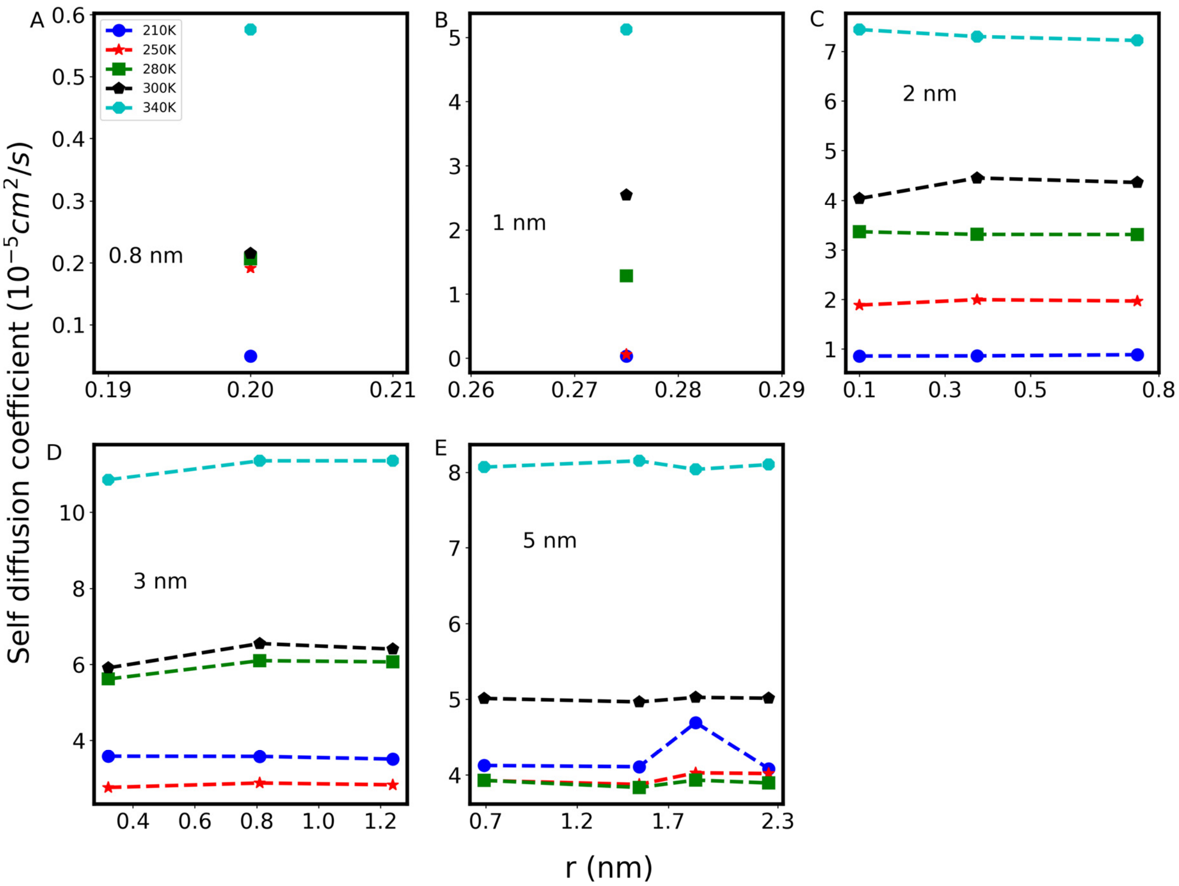

Figure S1. Self-diffusion coefficients of different water shells, shown in Figure 2 of the main text, inside different CNT sizes of A)0.8nm, B) 1.0nm, C) 2.0nm, D) 3.0nm, and E) 5nm. The x-axis is the distance of water shells from the center of CNTs. Colors represent different temperatures; Blue (210K), red (250K), green(280K), black (300K), and cyan (340K).
